# Automatic segmentation of coronary lumen and external elastic membrane in intravascular ultrasound images using 8-layer U-Net

**DOI:** 10.1186/s12938-021-00852-0

**Published:** 2021-02-06

**Authors:** Liang Dong, Wenbing Jiang, Wei Lu, Jun Jiang, Ya Zhao, Xiangfen Song, Xiaochang Leng, Hang Zhao, Jian’an Wang, Changling Li, Jianping Xiang

**Affiliations:** 1 The Department of Cardiology, The Second Affiliated Hospital of Zhejiang University School of Medicine, Hangzhou, China; 2 The Department of Cardiology, Wenzhou People Hospital, Wenzhou, China; 3 ArteryFlow Technology Co., Ltd., Hangzhou, China

**Keywords:** IVUS, Coronary, Segmentation, Lumen, EEM, MeshGrid, U-Net

## Abstract

**Background:**

Intravascular ultrasound (IVUS) is the golden standard in accessing the coronary lesions, stenosis, and atherosclerosis plaques. In this paper, a fully automatic approach by an 8-layer U-Net is developed to segment the coronary artery lumen and the area bounded by external elastic membrane (EEM), i.e., cross-sectional area (EEM-CSA). The database comprises single-vendor and single-frequency IVUS data. Particularly, the proposed data augmentation of MeshGrid combined with flip and rotation operations is implemented, improving the model performance without pre- or post-processing of the raw IVUS images.

**Results:**

The mean intersection of union (MIoU) of 0.937 and 0.804 for the lumen and EEM-CSA, respectively, were achieved, which exceeded the manual labeling accuracy of the clinician.

**Conclusion:**

The accuracy shown by the proposed method is sufficient for subsequent reconstruction of 3D-IVUS images, which is essential for doctors’ diagnosis in the tissue characterization of coronary artery walls and plaque compositions, qualitatively and quantitatively.

## Background

Coronary heart disease has been the leading cause of death worldwide [1], and the coronary atherosclerosis is the dominant cause of coronary heart disease. In early atherosclerosis, coronary artery remodeling slows down the progression of vascular stenosis with the accumulation of coronary plaques. Intravascular ultrasound (IVUS) is one of the most effective real-time medical imaging techniques, which plays a critical role in the diagnosis and treatment of coronary heart disease.

2D IVUS images acquired serially by an IVUS catheter pulling back through the coronary artery can evaluate arterial distensibility caused by atherosclerotic plaque. The accurate segmentation of lumen and external elastic membrane cross-sectional area (EEM-CSA) from 2D coronary IVUS images that contributes to assessing the atherosclerosis plaque and its vulnerability by measuring lumen diameter, plaque eccentricity, plaque burden, etc., has crucial clinical significance. However, it is time-consuming and experience-dependent for doctors to manually delineate the lumen and EEM contours on the 2D IVUS images. A typical IVUS pullback contains more than 3000 images, so an accurate, fast, and fully automatic segmentation of lumen and EEM-CSA is highly desirable, but remains a challenging task due to the relative complexity of the IVUS images.

Several segmentation techniques and methods in image processing and computer vision have been performed for coronary IVUS images [2, 3]. Traditional image processing methods, including graph search, active surfaces, and active contours, were applied to segment IVUS images based on local image properties or global gray-level properties [4]. 3D fast marching method [5, 6], incorporating the texture gradient and the gray-level gradient, was applied to segment the walls of the coronary artery with an interactive initialization on EEM borders. In recent years, deep learning has been widely applied in the medical imaging analysis and achieved remarkable results [7, 8]. It has been utilized to detect the lumen and media–adventitia borders in IVUS due to its capabilities in automatic feature extraction [9, 10].

In this paper, we develop a U-Net [11] and evaluate the modified U-Net-based pipeline that automatically segments the lumen and EEM-CSA from 2D IVUS images. The pipeline has two major steps: first, the data augmentation of MeshGrid combined with flip and rotation operations (MeshGrid–Flip–Rotate) is performed on raw IVUS images; second, an 8-layer deep U-Net is used for pixel-level prediction [12].

## Results

**Fig. 1:**
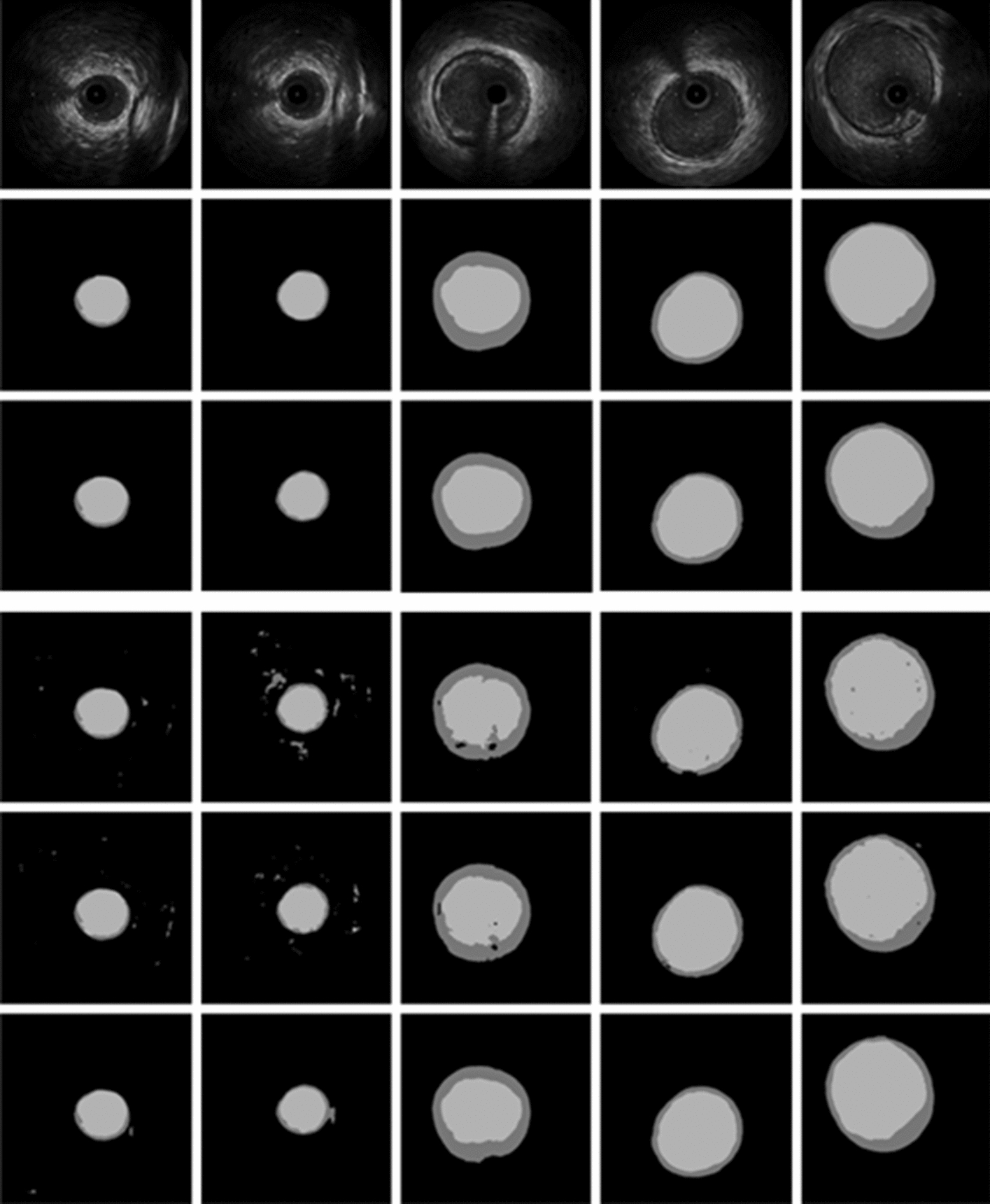
${6} \times {5}$ image matrix of segmentation result comparison for four different data augmentation strategies. The rows show the original image and different image augmentation strategies, including raw IVUS images as inputs (1st row), ground truth images as outputs (2nd row), No augmentation (4th row), Meshgrid (5th row), Flip–Rotate (6th row) and Meshgrid–Flip–Rotate (3rd row). The columns represent different IVUS image cases, choosing images of different shapes and sizes as much as possible (1st columns to 5th columns). The figure shows that Meshgrid–Flip–Rotate (3rd row) method have best segmentation performance, which are very close to the ground truth images

Experiments were carried out for segmenting the lumen and EEM-CSA with four augmentation strategies of No Augmentation, Flip–Rotate, MeshGrid, MeshGrid–Flip–Rotate. The contours predicted by the method with MeshGrid–Flip–Rotate augmentation (3rd row of Fig. 1) were in higher agreement with the ground truth (2nd row of Fig. 1) for a range of morphologies, in comparison with those with No augmentation (4th row of Fig. 1), MeshGrid (5th row of Fig. 1), and Flip–Rotate (6th row of Fig. 1). Taking the 2nd and 3rd column as example, the results of last 3 rows show that some noise points would be segmented in the background and EEM-CSA area.

Table 1 quantifies the segmentation results from the four data augmentation strategies. Compared with the other three data augmentation strategies, the way of MeshGrid–Flip–Rotate presented better segmentation performance in both lumen and EEM-CSA, with MIoU of 0.937 and 0.804, respectively. The reason why 8-layer U-Net does not exceed EEM segmentation result of Ji Yang et al. [10] could be addressed from two perspectives. On the one hand, the training data are too small to train a powerful segmentation model; for another, 8-layer U-Net’s architecture could be further optimized.

**Table 1:** The quantitative performance for the four different data augmentation strategies

Augmentation strategies	MIoU (lumen)	MIoU (EEM-CSA)
No augmentation	0.872	0.703
Flip–Rotate	0.915	0.759
MeshGrid	0.894	0.747
MeshGrid–Flip–Rotate	*0.937*	0.804
Ji Yang et al.[10]	0.900	*0.860*

The italic number results represent best MIoU performance in Lumen and EEM-CSA, our MeshGrid–Flip–Rotate can achieve 0.937 MIoU of lumen, which is better than other methods. But MIoU results of EEM-CSA is 0.804, which is inferior to 0.860 of Ji Yang et al.

## Discussion

The IVUS images varied significantly from the intensity gradient of edge of lumen to the contour curvature of plaques. The current dataset was limited to the single-vendor and single-brand. However, the proposed method provided acceptable segmentation results for both lumen and EEM-CSA from the frames in the dataset. On the visual comparison, in the case of the EEM-CSA segmentation, the performance was lower for complex frames while it was comparable good for simple frames; excellent results were seen for lumen segmentation for all cases. The segmentation of bifurcation images might be difficult due to the ambiguous vessel definition. The amount of calcified plaque could be possibly underestimated by IVUS and images of the vessel wall could be degraded due to geometric distortions, and shadows at the back of calcified plaques, all of these could make the model difficult to train. The case data including calcified plaque needs to be enriched in future to train a more robust model.

The data augmentation of MeshGrid–Flip–Rotate helped improve the segmentation performance. It was capable of generalizing well to eliminate the outliers (3rd row of Fig. 3). Neither pre-processing steps nor post-processing steps were necessary. The model was trained well on the current dataset, which provided MIoU of 0.937 for lumen predictions. This result is better than the results in the literature in Table 1. Investigating the reason, we believe that the help mainly comes from MeshGrid–Flip–Rotate data augmentation method and the 8-layer U-Net network that can extract more image features. However, when the testing set deviated far from the training set, such as serious artifacts, mixture plaques and branch vessels, the accuracy for EEM-CSA became relatively low (MIoU of 0.804). It can be improved largely when more coronary IVUS data of different categories are collected for training in the future.

From clinical perspective, a clinical threshold to assess the quality of the method should be provided by expert physicians to interpret the segmentation feature with complex or simple frames. For example, a fast pullback through the calcified lesion may result in loss of image features, increase of catheter artifacts and calcified shadows from the echogenicity of the lumen and plaque textures, which makes it tougher to be annotated even by experienced physicians. The cardiac cycle motion and coronary vessel pulsation due to the variability or arrhythmia of heart rate might push the catheter to touch the vessel and plaque boundaries, which increase the artifacts and motion jitters in the IVUS images.

Our future research is to extend the current dataset to enhance the robustness and generality of our method presented in this paper. The heterogeneous dataset of IVUS images shall cover different medical centers, different probe frequencies from different venders. More IVUS image categories from different artery pullback sections and different characteristics should be considered: plaque, bifurcations, branches, shadow artifact, stent, catheter artifact, etc. Each frame shall be cross-labeled by three expert physicians according to the respective categories to assess the method, which will make it more convincing.

## Conclusion

In this paper, an 8-layer U-Net is proposed with the data augmentation of MeshGrid–Flip–Rotate, which specifically fits for the coronary IVUS lumen and EEM-CSA segmentation task. The experimental results show its superiority in segmentation accuracy and efficiency. Furthermore, it provides a good start for the image-based gating to implement 3D-IVUS reconstruction when fused with X-ray projections, which enables fluid and dynamic analysis on plaques and vascular walls of coronary arteries.

## Method

In this section, we first introduce the coronary IVUS dataset used for training and testing. Then, the 8-layer deep U-Net architecture that predicts the masks for the lumen and the EEM-CSA of IVUS images is presented. The training details are described, and the metric for evaluating the proposed method is illustrated.

### Dataset and augmentation

We use the coronary IVUS dataset from The Second Affiliated Hospital of Zhejiang University School of Medicine. It consists of in vivo pullback of coronary artery acquired by the iLab IVUS from Boston Scientific Corporation equipped with the 40-MHz OptiCross catheter. It contains IVUS frames from 30 patients, which are chosen at the end-diastolic cardiac phase in DICOM formats, with the resolution of 512 × 512. The dataset is divided into two parts, 567 frames of 24 patients for training and 108 frames of 6 patients for testing, respectively. The training set is used for building the deep learning model and the testing set is used to evaluate the model performance.

**Fig. 2:**
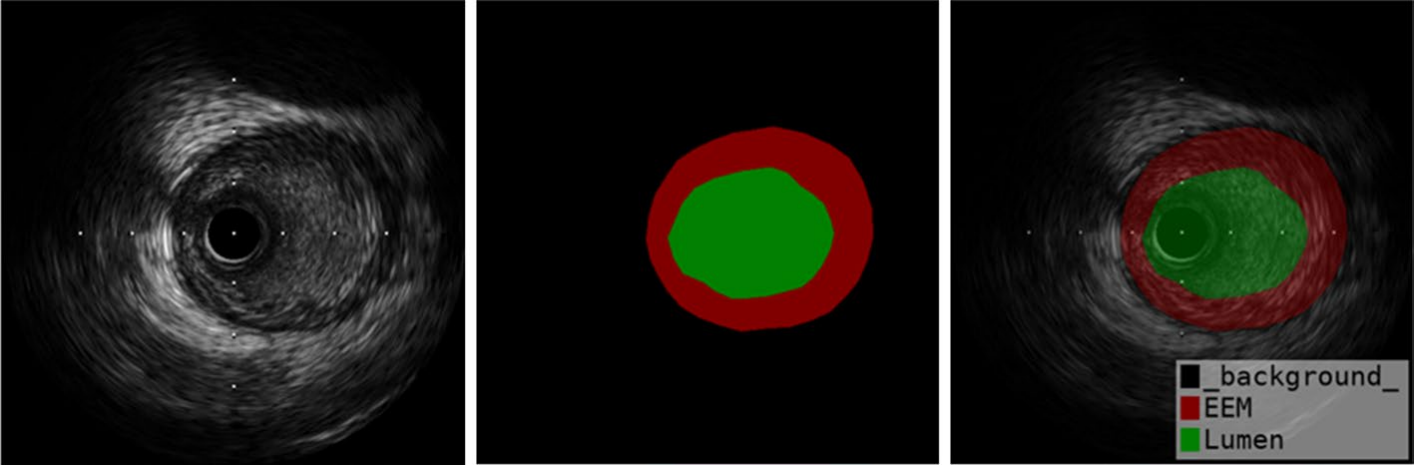
Ground truth labeling for lumen and EEM-CSA. The right image is raw IVUS image as train inputs, the middle image is annotation mask image as train outputs, the left image is a superposition of right and middle image

IVUS images contain catheter, lumen, endothelium, intima, media, external elastic membrane, adventitia, atherosclerosis plaque. The external elastic membrane is usually treated as the borders of media and adventitia. The media is gray or dark as it contains dense smooth muscle. The adventitia is similar to external tissues surrounding the vascular walls. The endothelium and intima are thinner than the lumen and media. Thus, the lumen and EEM-CSA can be manually annotated by experienced physicians as the ground truth for metric evaluation. Each IVUS frame has been manually annotated for the lumen and EEM-CSA in the short-axis view by three clinical experts, daily working with the specific IVUS brand from the Cardiology Department, shown in Fig. 2. Each expert is blinded to the other two experts’ annotations and each frame is repeatedly labeled by each of the three experts to ensure the correctness and blindness of the annotations. From the visual point of view of annotation, 92% of the annotated cases have high consistency.

The training set comprises 567 frames, which is not large enough for training a CNN model from scratch. Data augmentation is essential for better performance. The augmentation is twofold and performed online. First, the coronary IVUS raw images and the corresponding ground truth are randomly (1) rotated at angles: 90°, 180° or 270°; (2) flipped up–down or left–right. Secondly, the MeshGrid is added to the raw image at pixel-level, providing the relative location information. Due to the relatively fixed position like intima and adventitia in IVUS images, MeshGrid could play a good guiding role in training process, which guides the neural network where to look.

### Network architectures

The U-Net is one type of the fully convolutional network [13] and is the most common convolutional network architecture for biomedical image segmentation. It consists of encoder and decoder parts and predicts segmentation mask at pixel-level instead of image-level classification. The encoder part is used for down-sampling and extracts higher-level features. The decoder part is used for up-sampling the output from the encoder part and concatenates the feature maps of the corresponding layer by skip connection. The skip connection is to relieve the gradient diffusion problem due to deep layers. The final decoder layer is activated by softmax to produce the class probability map to recover the segment predictions.

**Fig. 3:**
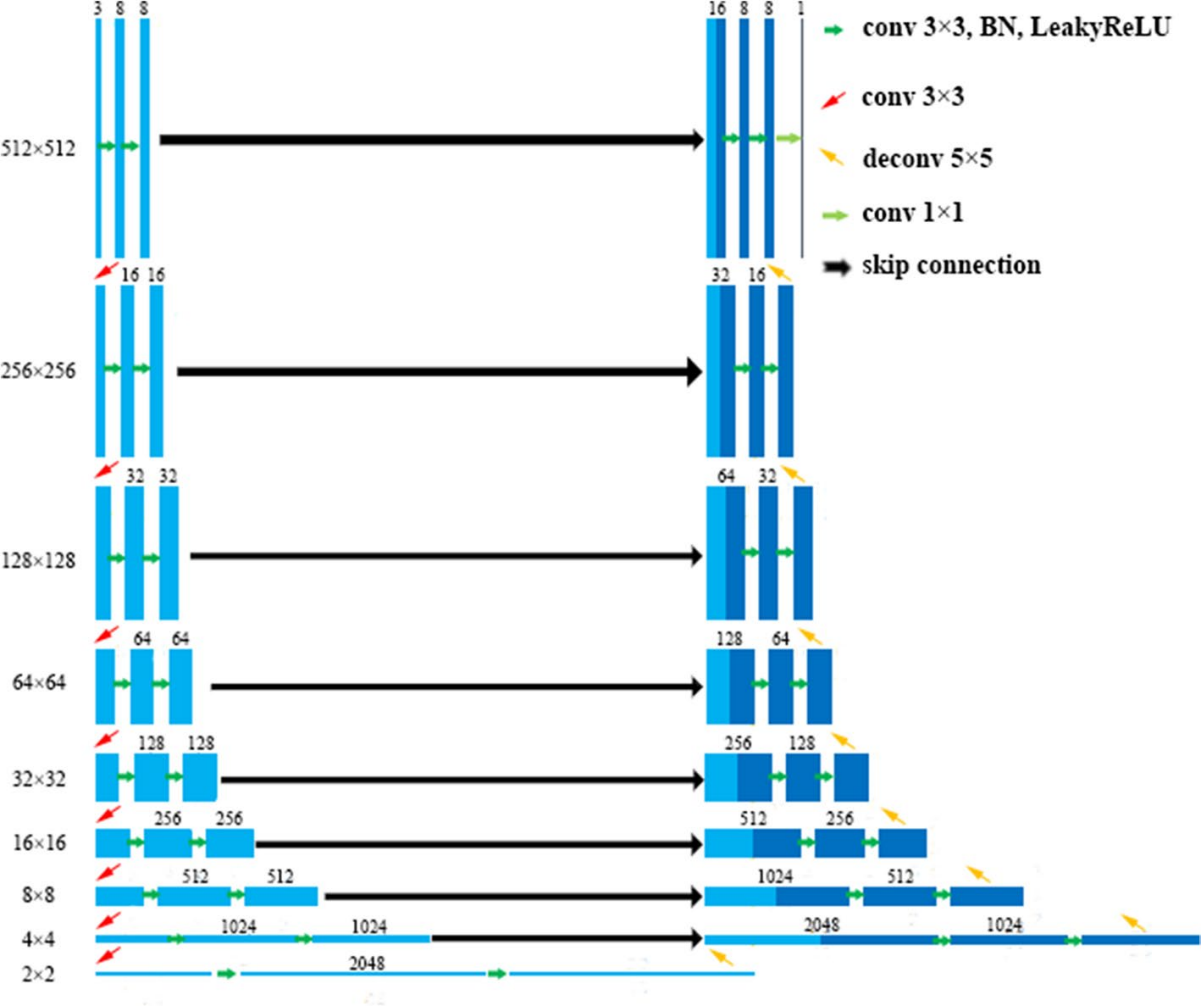
The proposed U-Net Architecture with 8 layers. The 8-layer deep U-Net consists of three parts, encoding networks (left in the figure), decoding networks (right in the figure) and skip connection (black arrow in the middle)

The encoder part has 9 blocks and each incorporates two repeated operations of 3 × 3 convolution, batch normalization and LeakyReLU activation. The down-sampling operation of 3 × 3 convolution with stride 2 × 2 reduces feature maps by half. The size of the 8th block is 2 × 2 to capture the deeper abstract information. The decoder part has 8 blocks to restore the image dimension. Each up-sampling operation contains a 5 × 5 deconvolution with stride 2. The skip connection concatenates the corresponding feature maps. The last convolution outputs the probability map of mask class prediction by softmax activation. The entire architecture is shown in Fig. 3. The parameter initialization of all layers of the model uses the random initialization method.

Compared to other U-Net variations, our proposed U-Net was no major innovation in structure. We replaced the original 4-layer network with an 8-layer network, which been able to extract deeper image features. The actual results also confirmed this simple deepening design.

### Implementation details

The model was trained and evaluated on Dell PowerEdge T640 server with Xeon Silver 4114 processor, 128 GB of RAM, and four Nvidia GTX 1080Ti graphics cards. It took less than 90 min for training and 10 ms per image for inference.

We implement model training with TensorFlow framework. The less frames are not enough to train the CNN from scratch, in addition to data augmentation, we also employed transfer learning to initialize the encoder of U-Net’s weights using VGG16[14]. The optimizer was Adam [15], which was fast and robust. The weights were initialized randomly and the batch size was set to 16. The initial learning rate was 0.001 with the decay of 0.1 every 2000 iterations. A total of 8000–10000 iterations were done for training. Lumen and EEM-CSA were trained and predicted at one shot with the softmax function as the output activation, which gave each pixel its class probability. The loss function was the sparse softmax cross entropy [16]:

1 $$L(p_{{\hat{y}}} ,p) = - \sum\limits_{j = 1}^{K} {p_{{\hat{y}_{j} }} \log (p{}_{j})} ,$$

2 $$p_{j} = {\text{softmax(}}x_{j} {)} = \frac{{e^{{x_{j} }} }}{{\sum\limits_{k = 1}^{K} {e^{{x_{k} }} } }},$$

with *K* being the number of classes, $p_{j}$ being the predicted probability belonging to class *j*, and $p_{{y_{j} }}$ being the true probability.

### Evaluation criteria

In semantic segmentation, the mean intersection over union (MIoU) is a widely used metric to evaluate the model, which is a common measure in semantic segmentation [17]. We compute the MIoU score between the ground truth and the predicted masks:

3 $${\text{MIoU}} = \frac{1}{k + 1}\sum\limits_{i = 0}^{k} {\frac{{p_{ii} }}{{\sum\limits_{j = 0}^{k} {p_{ij} } + \sum\limits_{j = 0}^{k} {p_{ji} - p_{ii} } }}} ,$$

with *k* being the number of classes excluding background, and $p_{ij}$ being the number of pixels of class *i* predicted to class *j*.

## Data Availability

The datasets analyzed during the current study are available from the corresponding author on reasonable request.

## Abbreviations

3D: Three-dimensional
IVUS: Intravascular ultrasound
EEM-CSA: External elastic membrane cross-sectional area
MIoU: Mean Intersection of Union
